# Defense Mechanisms and Repressive Coping Among Male Breast Cancer Patients

**DOI:** 10.3389/fpsyt.2021.718076

**Published:** 2021-12-10

**Authors:** Rainer Weber, Johannes C. Ehrenthal, Evamarie Brock-Midding, Sarah Halbach, Rachel Würstlein, Christoph Kowalski, Nicole Ernstmann

**Affiliations:** ^1^Department of Psychosomatic and Psychotherapy, Faculty of Medicine, University Hospital Cologne, Cologne, Germany; ^2^Department of Psychology, Faculty of Human Sciences, University of Cologne, Cologne, Germany; ^3^Center for Health Communication and Health Services Research, Department for Psychosomatic Medicine and Psychotherapy, University Hospital Bonn, Bonn, Germany; ^4^Center for Integrated Oncology (CIO Bonn), Bonn, Germany; ^5^Breast Center, Department of Gynecology and Obstetrics (Comprehensive Cancer Center Munich), University Hospital of Munich (Ludwig Maximilian University), Munich, Germany; ^6^German Cancer Society (DKG), Berlin, Germany

**Keywords:** defense mechanisms, male breast cancer, repressive coping, fear of progression, mixed-methods

## Abstract

**Objectives:** The concept of defense mechanisms has undergone extensive revision and expansion since Freud first described these processes. Initially formulated as an unconscious repression of unpleasant memories, with further development focusing on the role of defense mechanisms in the regulation of internal conflicts, the concept shifted and evolved to incorporate the adaptation to external demands, including intrapsychic and interpersonal handling of burden of illness. In addition to defense mechanisms, coping provides another perspective on human adjustment to difficult life events. While there is substantial research on both coping and defense mechanisms in various psychiatric and somatic diseases, including cancer, little is known about defensive regulation, coping, and their interaction in male breast cancer patients.

**Methods:** The present study is part of the N-Male project conducted between 2016 and 2018 in Germany (Male breast cancer: patients' needs in prevention, diagnosis, treatment, rehabilitation, and follow-up care). Semi-standardized interviews with 27 male breast cancer patients were analyzed with regard to defense mechanisms. In addition, fear of progression and repressive coping was assessed by self-report.

**Results:** There was considerable variety in levels of defensive functioning as well as repressive coping in our sample. We found no difference in overall levels of defensive functioning between men with vs. without repressive coping. However, patients with repressive coping demonstrated a decopupled association between fear of progression and defensive functioning as compared to patients without repressive coping.

**Discussion:** The study provides the first evidence of disease processing in male breast cancer patients Knowledge of patients' defense patterns and repressive coping seems promising for better planning targeted intervention strategies.

## Introduction

The observation that the mind has the ability to protect itself from confrontation with unpleasant thoughts and memories was first described by Freud in the context of his hysteria studies (1). Defense was mainly seen as a pathological phenomenon, intimately linked to what was called neurotic symptoms at that time (2). The concept of defense has undergone many transformations up to now, and is still losey linked to psychodynamic approaches to psychopathology and intervention (3). Defense mechanisms can be described as automatic psychological processes that can protect the individual from fear as well as other, more general demands and stressors. In other words: With the help of defense mechanisms, that usually operate outside of conscious planning and coping, individuals can modify the experience of internal, mainly emotional states as well as the processing of information from the external world.

By that, the concept of defensive functioning provides a framework for describing and understanding regulatory mental processes in mental disorders as well as life stressors or serious chronic stressors such as a cancer diagnosis. However, these expansions of the defense concept incorporate several important questions. For example, are some mechanisms more adaptive than others? Do individuals differ with regard to their common levels and mechanisms of defensive operations? At the core of these questions is the dual function of defenses, already pointed out by Anna Freud (4), which proposes that defensive functioning can be pathological, but protective and affect-regulating as well. Instead of distinguishing between normal and pathological defense mechanisms, she suggested that it should be noted whether there is a balance between different defense mechanisms. That is, whether other defense mechanisms are used, or only a few or only one, and how intensively the defense mechanisms occur. Anna Freud's approach was influential in advancing the discussion of the benefits or harms of defenses. These questions have been controversially discussed in the context of an assumed dichotomy of “adaptive vs. non-adaptive” defenses (5).

Cramer (6) supports the argumentation of Anna Freud. She describes that mental health does not necessarily have to be related to mature defense mechanisms. Instead, it depends on the flexible use of defense mechanisms in different situations. Various studies show that the use of immature forms of defense can be considered a risk factor for the development of psychopathology (7–9). Granier et al. (10) postulate that there is a compelling relationship between mental health and emotional flexibility. Findings from personality research support these assumptions. Mature defense mechanisms such as humor, altruism, and sublimation are associated with adaptive functioning (11, 12). With reference to this theoretical line of development, there is widespread agreement that defense mechanisms can be described with a hierarchical model from “immature” to “mature” defenses (7, 13, 14).

There has been intensive research on defensive functioning for several decades. In general, findings support a hierarchical model of groups of defenses being related to overall lower levels of functioning (3). According to Perry (15) research on defense mechanisms usually focuses on three different dimensions. The first dimension concerns the question of identifying defense mechanisms. Here, two different research traditions can be identified. On the one hand, the use of questionnaires (16) on the other hand the use of observer-rated measures (17). A second dimension relates to connections between defensive patterns and psychopathology, including personality disorders. For example, Perry et al. (18) examined four personality disorders (Schizotypical, Borderline, Antisocial, and Narccisstic). Immature defense mechanisms were central in all four personality disorders. Regarding specificity, there was for example a strong association between major image distorting defenses (e.g., splitting of self and other's images and the hysterical level defenses, dissociation, and repression) and borderline personality disorders. Another study by Hoglend and Perry (19) investigated the effect of defense mechanisms on the course of major depression. Here self observation on a high adaptive defense level at the beginning of psychotherapy was more often identified in those patients who improved more than predicted. The third dimension of research on defense mechanisms reflects on the question of the extent to which defense mechanisms can be changed in the course of psychotherapeutic treatment. Bond and Perry (9) reported about long term changes in defense styles with psychodynamic therapy for depressive, anxiety and personality disorders. In a recent study (20) it was shown that overall defensive functioning (ODF) and adaptive defenses increase over the course of treatment, whereas maladaptive and neurotic defenses did not change. A similar pattern was found by Schauenburg et al. (21) for individuals in inpatient psychotherapy. Here maladaptive defenses declined, adaptive defenses increased, while neurotic defenses remained stable. In a new review of changes in psychotherapies with personality disorders (22), defense mechanisms and an increase in insight play a crucial role for emotional and socio-cognitive change. In addition to the three dimensions of research on defense mechanisms described above, a branch of research has opened up in recent years that deals with the confrontation of serious illness, such as cancer.

In the meantime, the defense concept is also associated with newer concepts and clarifies the potential it has in describing human experience and behavior. For example recent findings on the relationship between mentalization, attachment, and defense mechanisms supports this. For example, patients with a secure attachment type showed a higher capacity for mentalization and the use of more mature defense mechanisms (23). At the same time, more research is needed on for example basic vs. applied aspects of defense mechanisms (3).

A broad conception of defense also incorporates other strategies for dealing with stressful, anxiety-producing demands and feelings from the research tradition of coping strategies. In contrast to defense mechanisms, coping strategies are traditionally understood to be used consciously and intentionally to restore mental equilibrium in the sense of an adjustment process (24, 25). The concepts of coping and defense originate from entirely different theories, namely psychoanalysis and stress theory. At the same time, especially concerning their regulatory aim, they might be regarded as two sides of the same coin. Several authors have discussed the relationship between coping and defense. For example, Haan (5) postulated a hierarchical relationship (coping—defense—fragmentation). However, the influential group around Lazarus did not make a distinction between the two concepts and sees defense merely as a particular case of a higher-level coping concept (26). For a more detailed discussion see for example (27). At the same time, the coping research adds a possibly valuable perspective to the study of defenses as it has defined several conditions that may describe or determine phenomena which could be relevant for the availability and display of defensive operations under certain conditions, such as repressive coping.

### Repressive Coping

The concept of repressive coping style goes back to Weinberger's et al. (28) seminal paper and has stimulated intensive research especially in behavioral medicine. Garssen (29) defined “repressive coping” as the tendency to inhibit the experience and the expression of negative feelings or unpleasant cognition in order to prevent one's positive self-image from being threatened (p. 471). According to Weinberger et al. (28), the phenomenon of repressive coping is best described by a combination of anxiety and defensiveness that occurs in response to a stress-inducing event. A total of three groups can be differentiated: the first group is characterized by low anxiousness and low social desirability. The second group contains individuals who are highly anxious but comparatively low in social desirability. The third group comprises individuals who show levels of social desirability but express low levels of anxiety. These groups differ on one crucial point. Individuals who express a low level of anxiety and a higher level of social desirability (i.e., repressors) show physiological reactions (heart rate, increased skin conductance) that are not compatible with subjective ratings of distress, indicating some kind of bio-psychological de-coupling. These individuals also do not show stress-induced feelings (29, 30). The early work by Weinberger and numerous later studies found that repressors dissociate their somatic reactions from their perceptions of distress, and in potentially stressful situations, report low levels of distress and anxiety but exhibit high levels of physiological activity (31). A number of studies indicate that repressors avoid negative affect (32, 33). A significant, but not surprising extension of the concept lies in observing that an individual's repressive coping style is associated with poor health and somatic illness. Similar findings have been demonstrated in cancer patients and patients with coronary heart disease (31). The results of a meta-analysis by Mund and Mitte (34) indicate a higher risk for repressive copers to be affected with at least one of several investigated diseases, but especially cancer and hypertension. However, the exact associations remain unclear, as well as the direction of the effect, i.e., whether repressive coping increases the risk for somatic diseases, or the other way round.

### Male Breast Cancer

Male breast cancer is a rare condition. Around just one percent of all breast cancer cases in the western world are diagnosed in men (35, 36). The rates increased slightly (1973–1998) as shown by Giordano et al. (37). The mean age at diagnosis is between 60 and 70 years. The awareness for breast cancer in men and the correct interpretation of related symptoms is very low. The presence of a painless lump is the most frequent indicator (38). Relatedly, symptoms are often diagnosed too late (39). Because of the comparatively low prevalence of the disease, many health care providers never encounter a male breast cancer patient (40). The treatment of male breast cancer patients is largely based on available evidence for females (41). Wang et al. (42) found that mortality after cancer diagnosis was higher among male patients with breast cancer compared to female breast cancer patients. This disparity persisted even when controlling for clinical characteristics, access to care, and specific treatment factors. A related problem is stigmatization. Most stigmatization concentrates on sexual stigmatization and ignorance of male breast cancer and mostly occurs in cancer care systems and work-related contexts. It seems that breast cancer is still seen as a “woman's disease,” as Midding et al. (43) pointed out in their paper. Emasculation (e.g., physical changes and changes in body image after treatment) can also lead to secondary stigmatization in the process of or after treatment. According to (44), most studies dealing with male breast cancer can be described as quantitative studies that focus on the disease's clinical aspects, such as risk factors, pathology issues, and treatment options. The authors criticize the focus on these purely medical aspects and call for more research attention to this disease's emotional challenges to men.

### Psychological Impact and Management of Breast Cancer in Woman and Men

In line with the lower prevalence rates, it is not surprising that knowledge about breast cancer's psycho-social factors is similarly lacking in men compared to a comparatively good database in female breast cancer patients (45, 46). Breast cancer can be a traumatic and stressful experience for women, but there are wide-ranging differences in the ways in which women respond and adapt to this disease (47). This study shows as well that income, cancer stage, fatigue and physical functioning are consistent predictors of adjustments. Psychosocial factors, such as optimism and anxiety, and perceived social support, coping strategies, and initial level of psychological functioning, were predictive of depression, anxiety, psychological distress, and quality of life in women with breast cancer. Lally and Brooks (48) were able to indicate the clinical usefulness of psychoeducational interventions that address the psychological adjustment needs of family members, spouses, and friends (supporters) who support women with early breast cancer in a systematic review. In line with these findings, Lewis et al. (49) reported that social support from family members and close friends is considered essential to be able to deal with the disease and its psycho-social consequences. France et al. (50) reported in a qualitative study that men prefer to talk exclusively to their wives. Similarly, effects of psycho-social support groups in women with breast cancer on coping with the disease and overall quality of life are well-documented (51). Again, research on the acceptance and effectiveness of appropriate support groups in men is lacking (44). According to Donovan and Flynn (52) female breast cancer (and associated psychological factors) is the most widely studied form of malignancy. It incorporates questions of coping with the disease, with cancer manifesting as a stressful life event in various ways, depending on type of disease, prognosis, and the subjectively experienced impairments.

How individuals adapt to and deal with the disease condition, however, is closely related to defense mechanism and levels of defensive functioning. The research literature has shown that psychoanalytically defined defensive processes can be regularly observed in dealing with a threat caused by cancer (e.g., the diagnosis, the consequences of the disease) (Di Guiseppe, 2020). Hartmann has pointed out the special function of defense mechanisms and thus made a significant contribution to connecting coping strategies and defense mechanisms. Defense mechanisms in cancer have the above mentioned dual function: on the one hand, they keep threatening feelings, memories and the like away from consciousness, and thus represent an adjustment performance. On the other hand defense processes also fulfill coping tasks. From our point of view, those two sides of the same coin are inseparably linked, and it is essential to emphasize the beneficial aspect of defense processes already described by A. Freud. For example, denial that might set in after being told of a severe diagnosis, such as cancer, would probably not be seen as pathological *per se*. Whether denial will have a negative effect on adjustment, role performance, or health behavior will probably show over time, for example concerning flexibility of defensive functioning, but also considering the ability to make use of other defense mechanisms and coping strategies as well. Accordingly, defense mechanisms can be understood as part of a meaningful adaptive effort that helps to regulate stressful life events such as cancer (53).

Empirically, Giese-Davis et al. (54) could show that defense mechanisms have a significant impact on health behavior and survival in breast cancer patients. Beresford et al. (55) demonstrated significant differences in survival probability in breast cancer patients, comparing mature and immature defense mechanisms: here, the use of mature defense mechanisms was associated with a significantly higher probability of survival. As described above, a hierarchy of defense mechanisms has been established in research on defense mechanisms (7, 56, 57). In a cross-sectional study comparing female breast cancer patients with a control group, Perry et al. (58) showed that female breast cancer patients used more immature forms of defense (e.g., denial, splitting, idealization) than participants in the control group. Di Guiseppe et al. (59) came to comparable results in a study of 145 newly diagnosed cancer patients. Cancer patients showed higher use of repression, suppression, rationalization, and lower use of affiliation, undoing, and devaluation of self-image compared to controls. Similar to findings reported above, it needs to be taken into account that this does not indicate a cause for cancer, but that a diagnosis of cancer itself is a life stressor that demands intensive defensive regulatory functioning. Unfortunately, it remains unclear whether all these findings can be generalized to male breast cancer patients.

From a perspective of defensive functioning and coping, what challenges do men face when diagnosed with breast cancer? Are there requirements for men that go beyond those for women with breast cancer? For example, analogous to the treatment of ductal breast carcinoma in women, adjuvant treatment with tamoxifen is the treatment of choice for male breast cancer patients. Despite the excellent tolerability, side effects can be described in the area of sexual dysfunction and loss of libido in men (60). Similarly, using a qualitative research design, Trusson and Quincey (61) addressed whether there would be differences between men and women in the experience of treatment-induced alopecia. It was found that both sexes experience hair loss as stressful, affecting gender identities and relationships. On the other hand, while men spoke more openly about the hair loss, preferably in a humorous manner, women tended to hold back in communicating about it and tended to hide the hair loss. Whether the diagnosis of breast cancer, which usually occurs in women, influences the perception of one's masculinity was examined in the study by Rayne et al. (62). No relationship was identified between affected masculinity and late presenting the symptoms in medical care. It must be noted that this finding is not in line with other studies that hypothesized a strong relationship between an affected masculinity and a delayed presentation of symptoms (52). Rayne et al. (62) discussed if the description of distress in the literature does not explain the difference between the burden of a cancer diagnosis (depression and anxiety) and specific problems with masculinity related to the fact of the male patients suffering from a woman's disease. There appears to be evidence that other factors may significantly impact the experience and management of male breast cancer than has been reported in the literature to date.

However, there are also similarities on the level of psychosocial burden in the face of cancer. For example, a significant concern for cancer patients is fear of cancer recurrence or fear of progression (63). While according to Pang and Humphris (64) related anxiety may be higher in women compared to men, there is again a research gap in male breast cancer:

To date, no study results are available on the occurrence and consequences of progression anxiety in men with breast cancer. In addition, to the best of our knowledge there is no study on defense mechanisms and their interrelationship with psychological variables in male breast cancer.

### Aims of the Study

Since the psychological consequences or processing in male breast cancer patients have been studied very little to none so far, we understand our research questions as in part descriptive, in part as hypothesis-testing, in part hypothesis-generating. As stated, to our knowledge, male breast cancer patients have not been studied on the topic of coping to date. Studies describing defense mechanisms in coping with the disease are entirely lacking. Likewise, studies dealing with the explanatory potential of repressive coping in male breast cancer patients are missing. Repressive coping may be an important aspect, as men with breast cancer face specific challenges in dealing with the disease, while at the same time experiencing a lack of support in the health-care system. Again, there is no research on patterns between defenses and repressive coping for this population.

This may be especially relevant in the context of dealing with cancer-related fears, such as fear of progression. Cancer patients in general have to cope with emotional distress like depression, anxiety, fear of progression of a life-threatening illness (65). For example Nakata et al. (63) could show in a sample of 927 female breast cancer patients that fear of progression was strongly associated with the need for psychological support (OR = 2.8). We therefore hypothesized that higher cancer-related fears triggers the use of defenses in men with breast cancer, whereas individual factors such as repressive coping may influence this assumed association.

Aims of the current study were therefore (1) To describe defensive functioning, repressive coping, and fear of progression in a sample of male breast cancer patients, (2) To describe patterns of defensive functioning in relationship to repressive coping in male breast cancer patients, and (3) To explore the possible impact of repressive coping on an association between fear of progression and defensive functioning in male breast cancer patients.

## Materials and Methods

The present study is part of the N-Male project conducted between 2016 and 2018 in Germany (Male breast cancer: patients' needs in prevention, diagnosis, treatment, rehabilitation, and follow-up care). The project was funded by the German Cancer Aid and approved by the Ethics Committee of the Medical Faculty of the University of Bonn (Reference Number: 087/16). The N-Male project is a cross-sectional observational study with a mixed-method approach (semi-standardized patient interviews and focus groups with health care providers and a standardized quantitative survey with different questionnaires before the interviews. Further information on the methods and results of the project is described elsewhere (40, 43, 66, 67).

### Sample and Study Design

The sample was composed in the sense of a non-probabilistic procedure according to the concept of theoretical sampling (68). Participants in the project were recruited nationwide through certified breast cancer centers, members of the Men with Breast Cancer network (Netzwerk Männer mit Brustkrebs e.V.), and invitations through newspaper advertisements. All participants had a confirmed breast cancer diagnosis (ICD-10 C50x or D05.X) and provided informed consent. One hundred men completed the quantitative survey, and a sub-sample of 27 participants was selected for the qualitative interviews according to purposeful sampling (69). Two female project staff members (Ph.D. candidates) conducted the interviews. Both project staff members were trained with the interview and prepared for possible difficult interview situations (emotionality and gender role conflicts) by an experienced psychotherapist. The interview guide was developed in an interdisciplinary expert group consisting of scientists, a patient representative, a representative from the health care provider group, and a psychotherapist. The semi-standardized interview guide offered open-ended narrative prompts and the opportunity to ask follow-up and in-depth questions. Thematically, the questions referred to each step in the treatment process (diagnosis, treatment phase, rehabilitation, and aftercare). The interviews were audio-recorded (after written consent was given) and transcribed according to uniform transcription standards. Interviews were conducted in the patient's preferred environment. In most cases, they were conducted at the participants' homes.

### Defensive Functioning

The Defense Mechanisms Rating Scales (DMRS) is a quantitative observer-rated method (70), which was included in the Appendix in DSM-IV (56). Thirty defense mechanisms based on a hierarchical organization are part of this qualitative tool assigned to 7 different levels (High-adaptive, Obsessional, Neurotic, Minor image distorting, Disavowal level, Major image distorting, and Action level (the Psychotic defense level was not used in this study). In addition, these levels can be further specified in three defensive categories (Mature Defensive Category, Neurotic Defensive Category and Immature Defensive Category) (see Table 1).

**Table 1:** DMRS Hierarchy of defense categories, levels, and individual defense mechanisms adapted from Perry et al. (71).

**I. Mature**
7 High adaptive Level (Mature): affiliation, altruism, anticipation, humor, self-assertion, self-observation, sublimation, suppression
**II. Neurotic**
6 Obsessional Level: intellectualization, isolation of affect, undoing
5 Other neurotic level: repression, dissociation and reaction formation, displacement
**III Immature**
4 Minor-image distorting level: devaluation of self or object images, idealization of self or object images, omnipotence
3 Disavowal Level: denial, projection, rationalization
2 Major image-distorting level: splitting of other's images, splitting of self-images, projective identification
1 Action level: acting out, hypochondrias, passive-aggression

Three different scores can be derived from the ratings: Individual defense score (an individual score calculated on the occurrence of defense used in a therapy session or an interview). Defense level score (their general level of adaptiveness hierarchically organizes the defense mechanisms. Each level can be calculated based on the used defenses). Overall Defensive Functioning (the ODF is obtained by taking the average of each defense level score. Its order weights this score in the hierarchical organization from 1—lowest to 7—highest), adapted from Perry et al. (71). Perry and Henry (72) proposed approximate reference scores for Overall Defensive Functioning Score (ODF) in clinical samples: “(1) Scores below 5.0 are associated with personality disorders or acute depression; (2) Scores between 5.0 and 5.5 are associated with the neurotic character and symptom disorders: (3) scores from 5.5 to 6.0 are associated with average healthy neurotic functioning, while (4) scores above 6.0 are associated with superior healthy-neurotic functioning” (p. 176).

### Repressive Coping

#### Marlowe Crown Social Desirability Scale

According to Weinberger et al. (28), repressive coping is assessed by using two different scales. One scale that measures manifest anxiety and another scale that measures defensiveness. This study's construct defensiveness is measured with the German 23-item version of the Marlowe Crown Social Desirability Scale (73, 74). The items, which can be answered with yes or no, refer to two different dimensions: socially desirable behavior that is, however, instead to be expected as unlikely (e.g., “No matter who I'm talking to, I'm always a good listener.”) and to socially undesirable behavior that is, however, very likely (e.g., “I like to gossip at times.”). The more often socially desirable but not expected events/items are answered with yes, and events/items characterized by openness are negated. The greater the tendency to present oneself with a socially desirable, idealized self-image.

#### State-Trait-Anxiety Inventory

The STAI is a self-report questionnaire designed to assess anxiety effects (75). With 20 items each, a four-point Likert scale assesses current anxiety (state) and general anxiety (trait). The trait version was used in the study to capture the cross-situational level of anxiety. The results from both self-description instruments (manifest anxiety and defensiveness) are combined into one score (repressive coping). The repressive coping style is based on the median score of both scales. Respondents with an anxiety score (STAI) below the median and a defensiveness score above the median (Marlowe-Crown Desirability Scale) are classified as repressors. Non-repressors can be divided into three different groups, according to Weinberger et al. (28). Low-anxious individuals (with anxiety and defensiveness scores below the median), high anxious individuals (with anxiety scores above and defensiveness scores below the median), and defensive individuals (with anxiety and defensiveness scores above the median). Besides classifying non-repressors into three groups, it is also possible to make a dichotomous classification (repressors and non-repressors) (76). Due to the small number of patients, we chose this classification for the analysis. Following Wiltink et al. (77) the main critic refers to the use of a median split to classify a person as repressor or non-repressor. However, the classificatory system has been replicated in numerous studies (78).

### Fear of Progression

The short form of the Fear of Progression scale (FoP-Q-SF) is a self-report instrument for identifying and assessing progression anxiety (79). A total of 12 items (5 point-Likert scales from 1 = never to 5 = often) are spread over four dimensions: affective reactions, partnership/family, occupation, and loss of autonomy. Scores range between 12 and 60 points. Herschbach et al. (80) proposed a cut-off score. A dysfunctional level of fear of progression is indicated if patients score higher than 33 points.

### Procedure and Analysis

Two trained raters with a longstanding expertise in psychodynamic constructs and clinical assessment (RW, JCE) evaluated the transcribed interviews. First, five interviews were rated separately. The results were discussed and compared. Because of the low number of interviews and complexity of the instrument, we did not strive to formally calculate indices of rater agreement. On the level of mechanisms and frequency, the agreement was high. Subsequently, the sessions were distributed between the two raters and evaluated separately. There were two meetings to discuss open questions without actually talking about rating levels to minimize rater drift. We also calculated interrater agreement on levels of specific defense mechanisms operationalized as frequencies of those mechanisms before agreement discussion. In total, 15 categories of defense mechanisms were rated in the interviews, with a high levels of agreement between both raters (*r* = 0.94, ICC_2.1_ = 0.95).

We used descriptive statistics, Pearson-correlations, and ANOVA to portrait distributions, and to assess associations and differences regarding the key variables. To test the moderating influence of repressive coping on the association between fear of progression and overall ODF level of defensive functioning, we conducted bootstrapping-based moderation analyses (number of bootstrap samples *M* = 5,000), which is especially robust given our sample size. To acknowledge the impact of possible covariates, we controlled for age and time since diagnosis as possibly relevant covariates. Those seemed especially relevant, as age may be a general factor influencing all variables, and time since diagnosis may be especially relevant for fear of progression. All analyses were conducted with IBM SPSS 25 and the PROCESS macro version 3.4 (81).

## Results

There were a total of 124 male breast cancer patients who wished to participate in the study. Of these 124 patients, three patients were excluded again because they did not meet the inclusion criteria. Some interested patients could no longer participate in the study due to the severity of the disease or death. A pre-test was conducted with four patients to field test the questions. One hundred seventeen patients were sent the questionnaire package. The response rate was 88.0% (*n* = 103). After reviewing the questionnaires (questionnaires with a proportion of missing values >30% were excluded from the analysis), 100 patients were included in the analysis (adjusted response rate 85.5%. Table 2 shows the socio-demographic and disease-related data of the sample. Data from the quantitative sample are in parentheses. Significant differences to the sample of 27 patients who were additionally interviewed could not be detected. One interview was conducted over the phone for logistical reasons. Unfortunately, there were repeated interruptions and poor recording quality, which made the interview incomparable to the other material, so it was excluded from the analysis.

**Table 2:** Socio-demographic and disease-related characteristics of the whole sample.

	***n***	**(*n*)**	**%**	**(%)**	**Mean**	**(mean)**	**Min**	**(min)**	**Max**	**(max)**
**Sociodemographic characteristics**										
Age in years [missing 1 (2)]					64.8	(66.91)	42	(39)	89	(89)
**Living with a partner [missing 3 (6)]**										
Yes	19	(82)	79	(87.2)						
No	5	(12)	20.8	(12.8)						
**Children [missing 1 (6)]**										
Yes	20	(79)	76.9	(84.0)						
No	6	(15)	23.1	(16.0)						
**Education (multiple answers possible) [missing 1 (2)]**										
No-schooling-leaving certificate	0	(2)	0	(2.0)						
Lower school-leaving certificate	11	(41)	42.3	(41.8)						
Intermediate school-leaving certificate	8	(27)	30.8	(27.6)						
General or subject-specific university entrance qualification	11	(35)	42.3	(35.7)						
**Diagnose related characteristics**										
**Types of treatment received (multiple answers possible) [missing 0 (0)]**										
Surgery	27	(97)	100	(97.0)						
Chemotherapy	16	(56)	59.3	(56.0)						
Radiation therapy	16	(65)	69.3	(65.0)						
(Anti) Hormone therapy	22	(75)	81.5	(75.0)						
I do not know	1	(2)	3.7	(2.0)						
**First diagnosis [missing 2 (4)]**										
Yes	24	(92)	96.0	(95.8)						
No	1	(4)	4.0	(4.2)						
Time since first diagnosis (in years) [missing 1 (5)]					4.1	(3.61)	<1	(<1)	17	(20)

*Qualitative sample N = 27; quantitative sample (N = 100). Numbers of quantitative sample in brackets*.

On average, participants in the study are in their sixth decade in both samples (quantitative and qualitative sample), ranging 39–89 years. Most of the patients live in a relationship and have children. Overall, the level of education is relatively high in both samples. Concerning the disease-specific data, almost all patients have undergone surgery, and chemotherapy was performed in about half of the patients. The proportion of patients who also underwent radiation therapy was high in both samples. Almost all patients were diagnosed with breast cancer for the first time, although the time window since diagnosis varied greatly, averaging just under 4 years.

### Defense Mechanisms in Male Breast Cancer Patients

Table 3 shows the mean values and standard deviations of the defense categories (Mature, Neurotic, and Immature). Male breast cancer males have a mean ODF value of 5.62 (SD = 0.82). However, to better understand the levels of different defense patterns in the group of male breast cancer patients, the ODF values can be related to clinical reference groups. About 30% of the respondents exhibited a mature form of defense organization in the transcripts (e.g., superior healthy neurotic functioning). In contrast, an almost equally large number of respondents reacted to the interview with defense patterns regularly found in patients with personality disorders (e.g., borderline) and depressive disorders (26.9%). The largest part of the sample, however, showed neurotic defense patterns in the interview.

**Table 3:** Overall defensive functioning and defense categories.

	**Mean**	**SD**
ODF (overall defensive functioning)	5.62	0.82
Mature	41.65	27.60
Neurotic	36.66	20.36
Immature	21.68	19.85

### How Is the Construct Repressive Coping Distributed?

In the analysis, 46.2% of the sample (*N* = 12) were classified as non-repressors and 53.8% (*N* = 14) as repressors. Both groups did not differ in relevant socio-demographic or disease-related data (age, marital status, disease duration). There was only one exception: patients classified as non-repressors had already had experience with breast cancer in their families (*X*^2^ (1, *N* = 26) *r* = 5.60, *p* < 0.05).

### Defense Mechanisms and Repressive Coping

**Figure 1:**
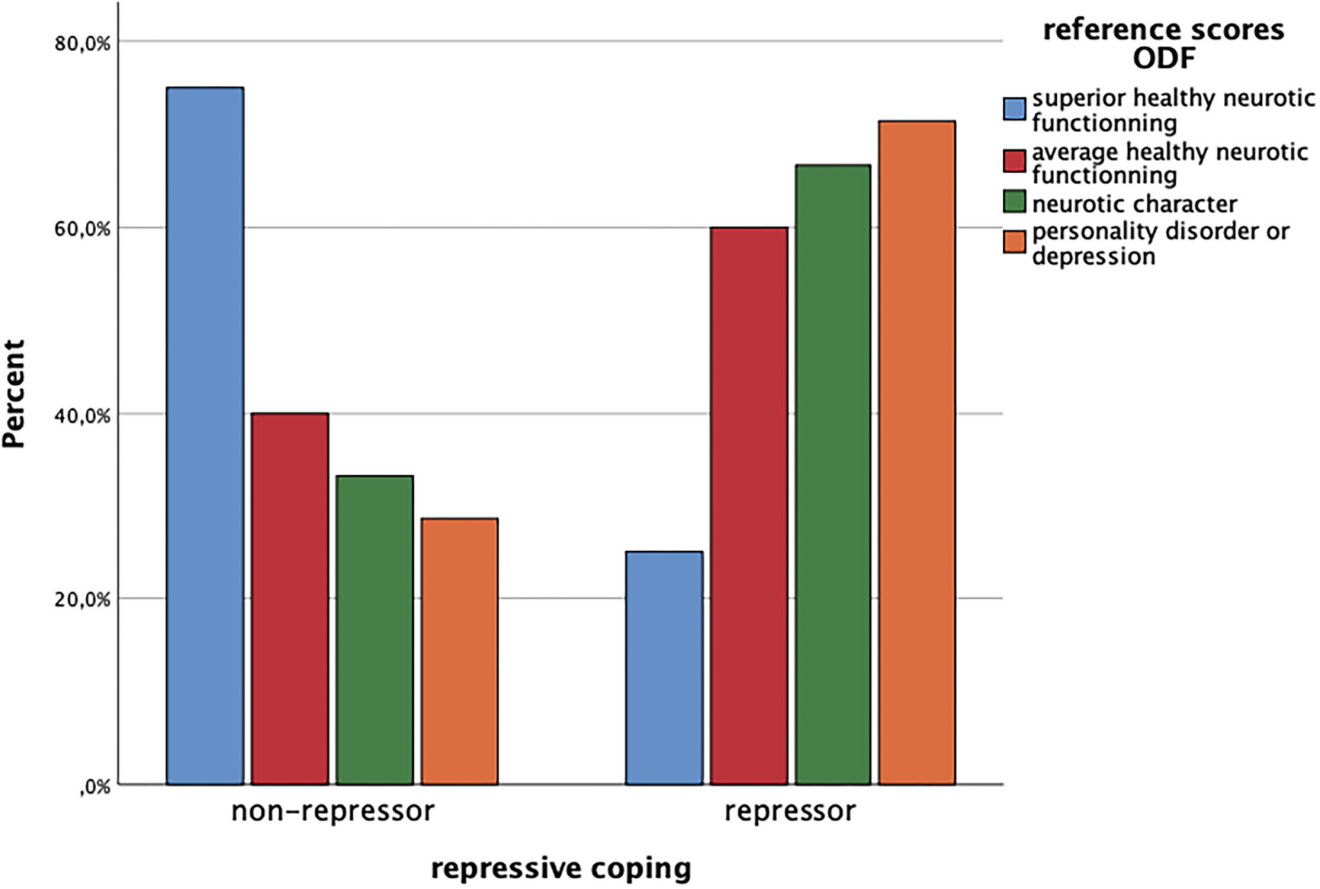
Reference scores ODF and repressive coping. ODF, overall defensive functioning categories of the Defense Mechanism Rating Scales (DMRS); FoP-Q-SF, Fear of Progression; repressive coping = category derived from Marlowe-Crown Social Desirability Scale and State-Trait-Anxiety Scale (STAI-Trait).

In a first step, we descriptively assessed the distribution of prototypical levels of defensive functioning in the group of repressive copers and non-repressors, and then tested differences in overall defensive functioning by means of ODF *via* ANOVA. While Figure 1 seemingly indicates a comparatively higher use of more mature defense patterns (superior healthy neurotic functioning) in the non-repressors than patients included in the group of repressors, concerning overall ODF there was no significant difference [*F*_(1, 24)_ = 3.40, *p* = 0.08] between non-repressors (*M* = 5.9, sd = 0.83) and repressors (*M* = 5.36, sd = 0.75).

### Associations Between Defense Mechanisms (ODF), Repressive Coping, and Fear of Progression (FoP-Q-SF)?

**Figure 2:**
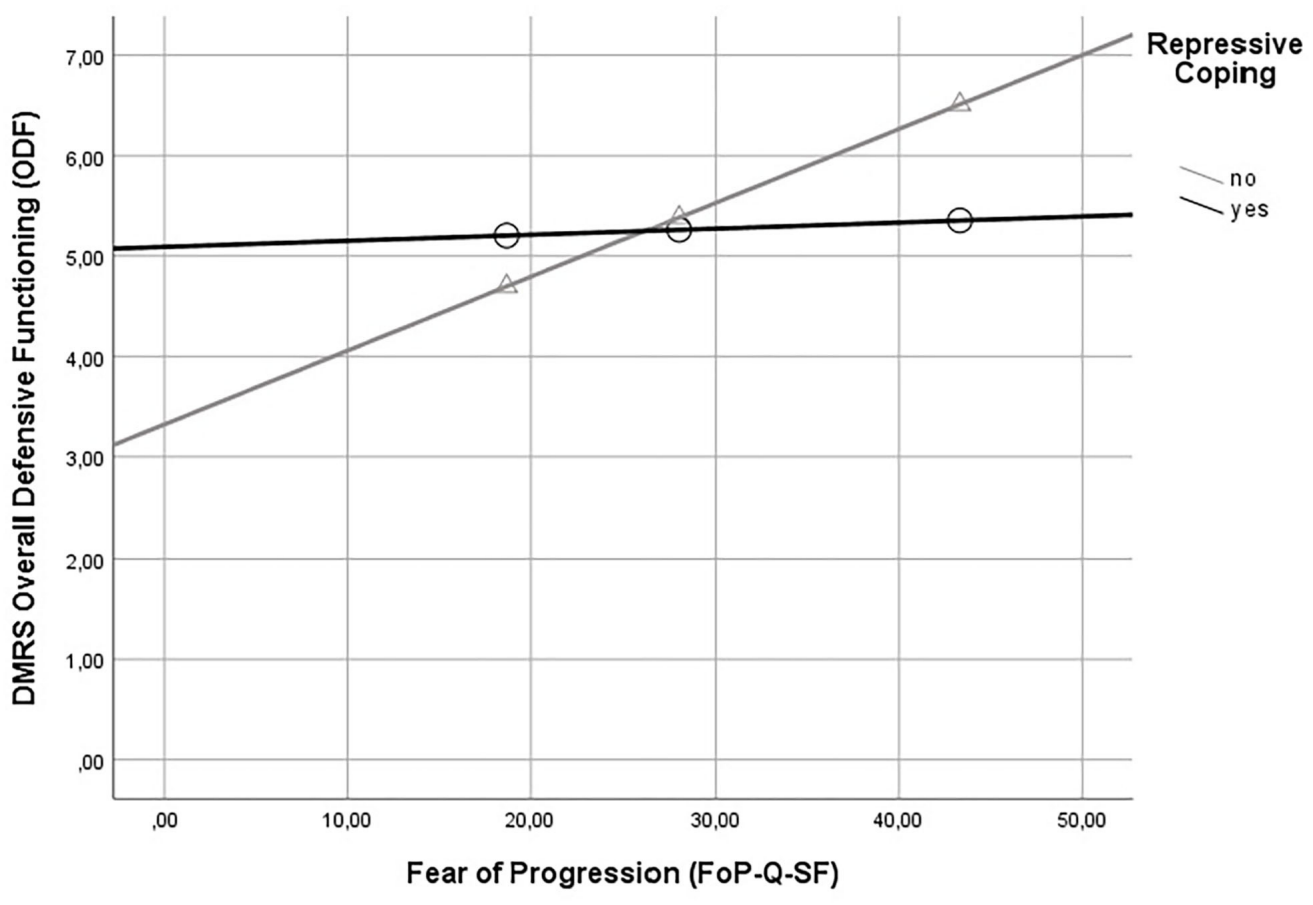
Moderation of the association between fear of progression and defensive functioning (DMRS) by repressive coping. DMRS, Defense Mechanism Rating Scales; FoP-Q-SF, Fear of Progression; repressive coping = category derived from Marlowe-Crown Social Desirability Scale and State-Trait-Anxiety Scale (STAI-Trait). DMRS overall defensive functioning (ODF) served as the dependent variable.

In addition to non-significant differences between repressors vs. non-repressors regarding ODF levels of defensive functioning, ODF was significantly associated with fear of progression (*r* = 0.43, *p* < 0.05). In other words, the higher the fear of a worsening of cancer, the higher levels of (more adaptive) defensive functioning. Regarding our hypothesis, bootstrapping-based analyses found that the association between fear of progression and overall defensive functioning while talking about cancer-related experiences was moderated by repressive coping. In other words, under conditions of no repressive coping, higher levels of fear of progression were associated with higher levels of (more adaptive) defensive functioning. Under conditions of repressive coping as a regulatory style, there was no association between both variables (see Table 4; Figure 2).

**Table 4:** Association between fear of progression and defensive functioning (DMRS) in male breast cancer patients with vs. without repressive coping.

**Variable**	**Coefficient**	***t***	**LLCI**	**ULCI**
FoP-Q-SF	0.08	2.79^*^	0.02	0.13
Group (Repressive coping yes/no)	1.77	1.66	−0.48	4.01
FoP-Q-SF × group	−0.07	−2.16^*^	−0.13	−0.002
**Covariates**				
Age	−0.001	−0.58	−0.04	0.02
Years since diagnosis	0.01	0.22	−0.08	0.09

*DMRS, Defense Mechanism Rating Scales; FoP-Q-SF, Fear of Progression; repressive coping = category derived from Marlowe-Crown Social Desirability Scale and State-Trait-Anxiety Scale (STAI-Trait). DMRS overall defensive functioning (ODF) served as the dependent variable*.

* *p < 0.05*.

## Discussion

According to the low incidence of male breast cancer, studies dealing with psychosocial factors are rare and usually have small case numbers (44, 50). To the best of our knowledge, this is the first study that has looked at the relationship between defense mechanisms and the concept of repressive coping and other psychological variables in men with breast cancer. Due to the absence of comparative data from other studies with males, the results must be discussed from an exploratory perspective. Di Giuseppe et al. (82) summarized the relevant literature in their systematic review on defense mechanisms in cancer patients. A total of 15 studies were included in the analysis. Studies involving female breast cancer patients were the most represented, including other cancer types (e.g., colorectal carcinoma and cervical carcinoma). It is important to note that the assessment of defense mechanisms was very heterogeneous (self-reported questionnaires, projective tests, and clinician-reported rating scale), and there was only one study that used the DMRS. The latter study (58) examined women with breast cancer; there were fewer adaptive and neurotic and more immature defense patterns than in the control group. These results are consistent with female breast cancer patients (83). In this study, the DMRS was also used to capture defense mechanisms. In the analysis, the full range of High-adaptive defenses (e.g., affiliation, humor, and suppression), Major Image distorting defenses (e.g., splitting of self-images, splitting of other's, and projective identification), and Action defenses (e.g., acting out and passive-aggression) were found in the interviews. The ODF score (Overall Defensive Functioning) as a measure of defensive mechanism maturity was below 5.0 for the sample (ODF = 4.70), corresponding to a relatively lower use of more mature defensive patterns. However, concerning comparing the ODF values of this sample with reference values of a clinical (psychiatric/psychotherapeutic) samples, defense mechanisms that are rather attributed to an immature defense organization cannot be described as maladaptive *per se*. This attribution could only be made if the patients had been diagnosed with psychopathological symptoms. As long as these data are not available, it must be assumed that some of the patients can only cope with the life-threatening disease and its consequences with difficulty and with the help of more immature defense mechanisms. This result is also in line with the theory of Anna Freud, according to which the question of whether a defense mechanism can be described as adaptive or maladaptive depends, among other things, on the time at which it occurs and its intensity. In addition, the average time between diagnosis and interview was about 4 years, ranging from less than a year to 17 years after diagnosis. In this case, a different state of threat can also be assumed. To better understand related questions, prospective studies would be needed that interview men at various points in the course of the disease since diagnosis.

The men interviewed in this study reacted differently to breast cancer diagnosis, subsequent treatment consequences (all patients of the qualitative part of the study underwent mastectomy), and impact on their social life. Mature defense patterns were more pronounced than in the samples with female breast cancer patients. Strikingly, no male patients showed evidence of Major image distorting defenses or Action defenses. Accordingly, the maturity of the defense organization was high with an ODF score of >5 (5.52). Since there are no studies to date that have recorded defense mechanisms in male breast cancer patients, only a comparison with female breast cancer patients or gender-mixed cancer samples can be made here. The ODF of our male sample is descriptively in the higher range of ODF compared to other studies that have investigated defense mechanisms in cancer. Di Giuseppe et al. (83) reported an ODF of 4.7 (SD = 0.54) in a sample of female breast cancer patients with a formal diagnosis of breast cancer within the past 2 months. Zimmerman et al. (84) studied a group of cancer patients (men and women with different cancers) at different points in the disease course. Patients who were actively undergoing radiotherapy had the lowest ODF (ODF = 5.05) comparatively, whereas cancer survivors and controls had significantly higher ODF values (5.32 and 5.63, respectively). These values also remained stable when controlling for gender.

It is certainly not possible at this point to infer about gender-specific differences in the use of defense mechanisms in breast cancer. While ODF scores in our male breast cancer sample was descriptively higher than for example in Di Giuseppe et al. (83), this again can be related to general differences in sample selection and requires further research. As in Di Giuseppe's study female patients with a formal breast cancer diagnosis within the last 2 months were included, the sample is more likely to be newly diagnosed patients, who are usually characterized by uncertainty and anxiety and basic questions of intervention and survival, which makes it difficult to compare with our sample consisting of men with breast cancer and a time since diagnosis from more than 1 to 17 years. The results of Zimmermann's study are also difficult to compare with the data from this study. Here, too, is a difference in defensive behavior, which is certainly associated with the degree of threat. Patients who have to face a more or less recent diagnosis and the resulting treatment measures tend to use more immature forms of defense compared to patients who have been diagnosed for a longer time (e.g., cancer survivors).

### Relation of Defense Mechanisms to Repressive Coping and Other Variables?

No significant direct association could be demonstrated between the constructs repressive coping and defense mechanisms (ODF). Thus, the result suggests that both concepts may be conceptually similar, but measure different phenomena (25). Freud's work on hysteria focused attention on a person's unconscious reactions to unpleasant, anxiety-provoking thoughts and feelings. Mund and Mitte (34) noted that the defense operations used (Freud called the process *repression*) were described as pathogenic (conversion neurosis in hysteria). Nowadays, it is clear that regulatory defensive mechanisms have an essential function to which an individual can “automatically” fall back in stressful situations. By that, defense mechanisms fulfill protective functions and can be considered an essential variable in affect regulation. In this respect, the results of this study show that belonging to the group of repressors or the group of non-repressor has a moderating influence on the handling of fear of progression, in terms of the maturity of the defense patterns expressed in the interview.

It is owed to medical progress and increasingly better psycho-social care that more and more cancer patients can be described as long-term survivors. Prognostic uncertainty plays a decisive role in the psychological experience of many patients (85). The results of our study are consistent with the results of other studies that generally focused on fear of recurrence or progression in cancer patients (female and male). Götze reports on a sample of 1,002 cancer patients (all cancers) 5 and 10 years after diagnosis. Levels of illness anxiety were higher in the 5-year cohort than in the 10-year cohort. Higher illness anxiety was associated with female sex, younger age, and elevated anxiety scores (86). In our sample, most patients were still below the 5-year mark after diagnosis. Accordingly, the level of anxiety expressed in the questionnaire was high. Patients classified as non-repressors showed a higher fear of progression. These patients seem to face their fears better, whereby they can fall back on more mature defense mechanisms than patients classified as repressors who do not want to acknowledge the threat. Their defense patterns can be described as correspondingly immature. In addition to the general call for further studies on psychosocial factors in male breast cancer patients, including comparative studies with female breast cancer patients already described, knowledge of patients' defense patterns and their expression in the area of repressive coping seems promising for better planning targeted intervention strategies.

### Strengths and Limitations

One strength of the study is the small, yet comparatively large and well-defined sample among this rare disease condition in men. Furthermore, we were able to report on a number of psychological variables for the first time in this clinical group, including defense mechanisms and repressive coping. Limitations relate for example to recruitment. Most participants in the study were recruited through the Men with Breast Cancer Network (Netzwerk Männer mit Brustkrebs e.V.), which does imply high levels of self-selection. Unfortunately, no figures are available on how long patients have been in contact with this network. However, it can be assumed that the information offered, the possibilities of exchanging information about the subjective experience of the disease at network meetings or private talks influenced the discussion of male breast cancer disease. An advantage of the study is undoubtedly also its disadvantage. More extensive studies are needed, especially comparative studies with female breast cancer patients. The gender effect in the processing of this threat of cancer has been shown in the results. In order to develop more targeted intervention measures for male breast cancer patients, this research desideratum is mandatory.

### Clinical Implications and Future Dimensions

The results of this study suggest that it should have been conducted much earlier. Men who suffer from this disease, which is quite rare compared to women, face a variety of challenges in addition to the threat of cancer. For example, men with breast cancer are co-treated in a more feminine setting specializing in treating women with breast cancer. This leads to the experience of stigma for some of the men affected (43). Therefore, the consideration of coping with the disease including the more conscious coping strategies and the more unconscious defense mechanisms seems to be very helpful for male breast cancer patients to recognize the distress and neediness, which may be hidden behind gender models. A better knowledge of the specific disease management could be followed by interventions, as early as possible. However, as already mentioned above, prospective controlled studies are needed for this purpose.

## Data Availability Statement

According to the patient consent form, data are not available for scientific use by others than the project group members. Please direct any enquiries to Rainer Weber, rainer.weber@uni-koeln.de

## Ethics Statement

The studies involving human participants were reviewed and approved by Ethics Committee Medical Faculty of the University of Bonn. The patients/participants provided their written informed consent to participate in this study.

## Author Contributions

RWe and JE analyzed the interviews for defense mechanisms. RWe and JE drafted the first version of the manuscript and contributed equally to the manuscript. EB-M and SH conducted the interviews. RWe and JE performed the statistical analyses of this study. NE and CK planned the study and revised the manuscript in detail together with RWü. All authors contributed significantly to the article.

## Funding

This project (N-Male) was supported by the German Cancer Aid under Grant No. 111742.

## Conflict of Interest

The authors declare that the research was conducted in the absence of any commercial or financial relationships that could be construed as a potential conflict of interest.

## Publisher's Note

All claims expressed in this article are solely those of the authors and do not necessarily represent those of their affiliated organizations, or those of the publisher, the editors and the reviewers. Any product that may be evaluated in this article, or claim that may be made by its manufacturer, is not guaranteed or endorsed by the publisher.
